# Neurodevelopmental Consequences of Maternal Diabetes: Autophagy and Spatial Arrangement of Hippocampal Neurons

**DOI:** 10.1111/cns.70518

**Published:** 2025-07-14

**Authors:** Saleheh Mansouri Boutegaz, Mohammad Reza Namavar, Mehri Shadi, Hamid Kabiri‐rad, Saeed Vafaei‐Nezhad

**Affiliations:** ^1^ Department of Anatomical Sciences, Faculty of Medicine Birjand University of Medical Sciences Birjand Iran; ^2^ Histomorphometry and Stereology Research Center Shiraz University of Medical Sciences Shiraz Iran; ^3^ Clinical Neurology Research Center Shiraz University of Medical Sciences Shiraz Iran; ^4^ Department of Anatomical Sciences, School of Medicine Shiraz University of Medical Sciences Shiraz Iran; ^5^ Cellular & Molecular Research Center Birjand University of Medical Sciences Birjand Iran

**Keywords:** autophagy, gestational diabetes, hippocampus, spatial arrangement, streptozotocin

## Abstract

**Background:**

Gestational diabetes mellitus (GDM) is a prevalent metabolic disorder that disrupts fetal central nervous system (CNS) development. This study investigates the effects of maternal diabetes on hippocampal structure and autophagy‐related mechanisms in neonatal rats, focusing on the PI3K/mTOR signaling pathway.

**Methods:**

Forty female Wistar rats were divided into three groups: control (CON), diabetic (STZ‐D), and insulin‐treated diabetic (STZ‐INS). Hyperglycemia was induced using streptozotocin, and offspring were analyzed at postnatal day 14 (P14). Histological evaluations of hippocampal structure were conducted using hematoxylin and eosin (H&E) staining, and neuronal damage was assessed with toluidine blue staining. Autophagy‐related gene expression (Beclin‐1, LC‐3, ATG‐7) and the PI3K/mTOR signaling pathway were examined using real‐time PCR.

**Results:**

Offspring from the STZ‐D group exhibited significant reductions in hippocampal volume and increased dark neurons in the CA1 and CA2 regions compared to the CON and STZ‐INS groups. Gene expression analysis revealed a marked downregulation of ATG‐7 and significant upregulation of PI3K and mTOR in the STZ‐D group, while Beclin‐1 and LC‐3 showed no significant changes. Insulin treatment mitigated these adverse effects, preserving hippocampal structure and reducing neuronal damage. In addition, the results of the Voronoi tessellation method showed that hippocampal neural cells depict a regular pattern in different subfields in all experimental groups.

**Conclusion:**

Maternal hyperglycemia disrupts hippocampal development by altering autophagy and activating the PI3K/mTOR pathway, contributing to neuronal damage. Insulin treatment during pregnancy can counteract these effects, emphasizing the importance of glycemic control. These findings highlight potential therapeutic targets for mitigating CNS impairments in the offspring of diabetic mothers.

## Graphical abstract

The study explores the impact of maternal diabetes on neonatal brain development, revealing significant weight reductions and alterations in brain structure, suggesting potential neurodevelopmental consequences and insulin treatment benefits.

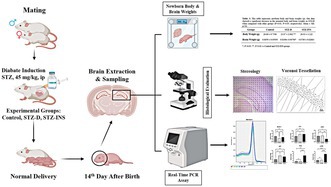

## 1 Introduction

Glucose intolerance during pregnancy is known as gestational diabetes, which is divided into pre‐gestational diabetes mellitus (PGDM) and gestational diabetes mellitus (GDM) [1, 2]. The global prevalence of diabetes during pregnancy is rising across diverse populations, influenced by factors such as age, race, ethnicity, and physiological characteristics, which could affect an estimated 5%–25% of pregnancies, of which 7.5% are associated with type 1 diabetes and 5% with type 2 diabetes [3, 4].

As diabetes during pregnancy is one of the most common metabolic disorders, numerous investigations indicated that excessive glucose levels might alter the intrauterine environment and could negatively affect both mothers and infants [5]. Gestational diabetes, a condition affecting organogenesis, embryogenesis, and fetal growth, increases the risk of developmental impairments in fetuses compared to healthy mothers, potentially increasing infant mortality and morbidity rates [6, 7].

Numerous studies have shown that the central nervous system (CNS) is highly sensitive to disruptions in glucose levels. Consequently, a range of neurodevelopmental, neurobehavioral, and neuropsychological disorders has been reported in the offspring of diabetic mothers [8, 9, 10]. In this regard, the results of a study conducted by Ornoy et al. [5] revealed that the children born to diabetic mothers have lower gross motor and Bruininks–Oseretsky fine (a test of Motor Proficiency) scores compared to children of non‐diabetic mothers. Moreover, they reported that these children showed a higher incidence of inattention and attention deficit hyperactivity disorder (ADHD) and a lower verbal intelligence quotient (IQ) [5, 11]. Furthermore, other studies have indicated that diabetes during pregnancy may adversely affect learning and memory abilities in offspring [11, 12]. Nowadays, it is well accepted that the hippocampal formation is the major part of the CNS, which plays a pivotal role in learning and memory formation in humans and rodents. This C‐shaped structure resides in the temporal lobe and comprises distinct subfields, including CA1, CA2, CA3, and the dentate gyrus [13]. Metabolic disturbances from diabetes during pregnancy could impair the development of different parts of the embryonic central nervous system, especially the hippocampus, which could lead to both structural and functional deficits at the cellular and molecular levels [11]. Prior research has shown that uncontrolled glucose levels during pregnancy can reduce hippocampal volume and increase the number of apoptotic cells and apoptotic‐related gene expression. Additionally, maternal diabetes has been reported to adversely impact synaptogenesis in the hippocampus and cerebellum by altering the expression levels of synaptophysin [12, 14].

Autophagy is a vital cellular process that maintains intracellular balance by repairing or auto‐digesting dysfunctional organelles and proteins and also plays an important role in many physiological processes, including development, aging, immunity, and brain function [15]. Many environmental and physiological situations can induce autophagy, which is mainly a reaction to stressors that disrupt cellular homeostasis, including nutrient deprivation, oxidative stress, hypoxia, and endoplasmic reticulum stress [16]. It is interesting to note that autophagy has a dual role in the nervous system; one of them could increase cell survival, while the other may promote cell death [17, 18]. There is growing evidence that demonstrates dysfunctions in the autophagy system have been observed across a range of neurodegenerative diseases such as bipolar disorder, schizophrenia, Parkinson's disease, Huntington's disease, and Alzheimer's disease [19, 20, 21].

This study investigates the impact of maternal hyperglycemia on hippocampal morphology and autophagy‐related gene expression in neonates by focusing on the AKT/PI3K/mTOR pathway.

## 2 Materials and Methods

### 2.1 Animals

To accomplish this study's objectives, 30 mature virgin female Wistar rats weighing approximately between 250 and 300 g body weight (6–8 weeks old) were utilized. All animals were acquired from the Research Centre of Experimental Medicine of Birjand University of Medical Sciences (Birjand, Iran). The animals were housed individually in cages under standardized conditions, including a controlled temperature of 21°C–23°C, 50%–60% humidity, and a 12‐h light/dark cycle. They were provided with unrestricted access to food and water throughout the experiment. It should be noted that all animal procedures were conducted in compliance with the Guide for the Care and Use of Laboratory Animals of Birjand University of Medical Sciences, Birjand, Iran (Ethical code: IR.BUMS.REC.1402.503).

### 2.2 Treatment

All experimental animals were randomly divided into three separate groups as follows:

1. Control (**CON**, *n*: 10).
2. Diabetic group (**STZ‐D**, *n*: 10).
3. Diabetic treated with insulin group (**STZ**‐**INS**, *n*: 10).

A single intraperitoneal injection of streptozotocin (45 mg/kg body weight) (Sigma–Aldrich Inc., Saint Louis, Missouri, USA) freshly dissolved in cold PBS (pH 4.5) was used to induce diabetes in the STZ groups.

The diabetic rats in the STZ‐INS group received protamine‐zinc insulin (NPH) (EXIR Pharmaceutical Company, Iran) administered subcutaneously at a dosage of 6 U/day, divided into 2 U at 8:00 AM and 4 U at 5:00 PM, as previously outlined [12]. The STZ‐D and CON groups were instead given subcutaneous PBS injections at the same times and in equivalent volumes. One week after initiating treatments, the female rats were paired with non‐diabetic males for overnight mating. The appearance of a vaginal plug the next morning was identified as the first day of pregnancy (gestational day 1, GD1) [12].

The animals' blood glucose concentration (BGC) was measured using a digital glucometer (BIONIME, Switzerland). For this study, only STZ‐treated female rats with BGC higher than 350 mg/dL were included on the day of plug detection and the day of delivery.

### 2.3 Tissue Preparation

All experimental animals were permitted to give birth normally, with the day of delivery designated as postnatal day 0 (P0). To eliminate the potential influence of milk from diabetic mothers and focus solely on the fetal environment, newborn rats from diabetic and insulin‐treated diabetic mothers were raised by control mothers.

On the 14th day (P14) after birth (P0), the pups' BGC and body weight were measured (*n* = 10 pups per groups). Then, the neonatal rats were deeply anesthetized and sacrificed by cervical dislocation. Immediately after that, their brains were carefully extracted, weighed, and immersed in the fixative solution (4% paraformaldehyde and 0.1 M glutaraldehyde in 0.1 M phosphate buffer, pH 7.4) at 4°C for 2 days. The brain samples were processed by standard histological methods and embedded in paraffin wax blocks. Afterward, coronal sections were obtained from brain tissue samples (10 μm‐thick) by using a sliding microtome (Litze, Germany). Finally, H&E and toluidine blue staining were carried out for histological goals. It should be mentioned that all histological analyses, including stereological volume estimation and dark neuron quantification, were performed by an investigator blinded to the group assignments to minimize observational bias.

### 2.4 Histological Evaluation

#### 2.4.1 Stereological Estimation

Based on previous studies, we used hematoxylin and eosin (H&E) staining, and hippocampal volume was estimated using the Cavalieri principle with systematic random sampling. Following fixation and tissue processing, brains were coronally sectioned at a thickness of 10 μm, and one section was selected every 100 μm throughout the entire rostro‐caudal extent of the hippocampus. On average, 10 sections per animal were analyzed (6 animals per group). A stereological point‐counting grid was superimposed on each image, with points spaced at regular intervals corresponding to a defined area calibrated to the microscope magnification. All volume estimations were performed by an investigator blinded to group allocation. The following formula was used to calculate the hippocampus' volume:

V=Σp.a/p.t

Σ*p* = total points toward hippocampus sections, a/*p* = area connected to each point, *t* = distance between sections sampled [22, 23].

#### 2.4.2 Evaluate the Number of Dark Neurons

In order to calculate the number of dark neurons, we performed toluidine blue staining. Briefly, slides were immersed in a staining solution containing 1% toluidine blue and 1% sodium borate and incubated overnight at room temperature. After staining, (5 rats/group, 4 fields/animal) 20 fields of hippocampus slides in each neonate were selected and scanned under a light microscope (Euromex‐CMEX‐10, Netherlands) to count the number of dark neurons in the hippocampal subregions (CA1, CA2, CA3, and DG) [24].

#### 2.4.3 Pattern Analysis and Spatial Distribution of the Neurons

The Voronoi tessellation approach was used to assess the spatial organization of neurons in the all‐hippocampal subfields in the various groups. The areas where the neurons are gathered and located nearby are covered by each polygon. Therefore, the area of a polygon represents the space occupied by a single neuron. Microscopic photographs of the randomly collected fields were used to create Voronoi polyhedron diagrams for each sub‐region of the neonatal hippocampi. A 40× objective lens was used to take the microscopic slide of the hippocampal slices, which were then loaded into Image J software (NIH, Bethesda, MD, USA). Following picture scale adjustments, the cellular profiles must be carefully classified as either glial or neuronal cells. The neurons were then marked by clicking on their nucleus. The mean areas of Voronoi polygons (μm^2^) were computed after this application was applied to the polygons. The spatial distribution of neurons is indicated by the coefficient of variation, or CV (standard deviation of the polygon areas/mean × 100). A distribution of neurons that falls between 33% and 64% is considered random, one that is less than 33% exhibits a regular pattern, and one that is more than 64% is regarded as clustered [25].

### 2.5 Molecular Assessment

#### 2.5.1 RNA Isolation and cDNA Synthesis

The whole RNA was isolated from the newborn rats' hippocampus samples (at P14) using the TRIzol solution (RNX‐Plus, Sinaclon, Iran) in accordance with the instructions supplied by the manufacturer. Subsequently, we used a commercial kit (Easy cDNA Synthesis Kit, Parstous) to synthesize cDNA from isolated RNA at 42°C for 60 min, following the manufacturer's instructions. Finally, cDNA samples were stored at −20°C.

#### 2.5.2 Real‐Time Polymerase Chain Reaction (Real‐Time PCR)

In this study, gene expression analysis of autophagy‐related genes (Beclin‐1, ATG‐7, LC‐3) and the key autophagy regulatory signaling pathway (AKT/PI3K/mTOR) was performed using a real‐time PCR system (Applied Biosystems Step One Instrument) with SYBR Green Master Mix and specific primer sets. Real‐time PCR experiments were conducted using coded RNA samples, and the individual performing the analysis was blinded to the experimental groups. It should be noted that primer sets were designed using sequences obtained from the NCBI database and verified for specificity through the NCBI BLAST tool (https://www.ncbi.nlm.nih.gov/tools/primer‐blast/) (Table 1).

**TABLE 1:** Primer sequences used for gene expression analysis, sequence (5′ → 3′).

Gene name	Primer sequences
Beclin‐1	F: AGCACGCCATGTATAGCAAAGA R: GGAAGAGGGAAAGGACAGCAT
ATG‐7	F: AGAAGAAGTTGAACGAGTAT R: CAGAGTCACCATTGTAGTAA
LC‐3	F: GGTCCAGTTGTGCCTTTATTGA R: GTGTGTGGGTTGTGTACGTCG
AKT	F: TATGAGAAGAAGCTGAGCCCAC R: CACACTCCATGCTGTCATCTT
PI3K	F: TCATGGATGCTTTGCAGGGT R: TGCCGTAAGTCATCGCCATT
mTOR	F: CAGAGATACGCCGTCATTCC R: TCAGAGTCAGGTGGTCATAGTC
Β‐Actin	F: TCCGTAAAGACCTCTATGCC R: GATAGAGCCACCAATCCACA

### 2.6 Statistical Analyses

In this study, all statistical analyses were performed using GraphPad Prism software, version 10. We assessed the normality of data distribution using the Shapiro–Wilk test for each experimental group. For datasets that met the assumption of normality (*p* > 0.05), we applied one‐way ANOVA followed by Tukey's post hoc test where appropriate for multiple group comparisons. For datasets that did not exhibit a normal distribution (*p* < 0.05), we used the Kruskal–Wallis test followed by Dunn's post hoc test for multiple comparisons. Data are presented as mean ± SD, with *p* < 0.05 considered statistically significant.

## 3 Results

### 3.1 Maternal and Neonatal BGCs

Maternal and neonatal blood glucose concentrations (BGCs) were assessed at specific time points, and the findings are presented in Table 2. At the first (GD1) and last days of pregnancy (GD21), a significant elevation in maternal BGCs was observed in the STZ‐D group compared to the control and STZ‐INS groups (****p < 0.0001, ^*####*^ *p* < 0.0001, respectively). These results confirm that hyperglycemia was sustained in diabetic rats throughout gestation.

**TABLE 2:** This table represents maternal and neonatal BGCs. Our data showed a significant increase in the maternal BGCs in the STZ‐D animals (*p* < 0.0001). In addition, at P0, BGCs in pups born to diabetic mothers were considerably higher than in the two other groups (*p* < 0.0001), while there were no significant differences at P14 in BGCs between all experimental groups. Mean ± SD.

Groups	Maternal blood glucose (mg/dL), *n* = 10		Pups' blood glucose (mg/dL), *n* = 10	
	GD1	GD21	P0	P14
Control	120.8 ± 8.4	119.6 ± 10.8	91.1 ± 6.3	135.8 ± 13.8
STZ‐D	444.6 ± 74.1^a^, ^b^	419.6 ± 61.2^a^, ^b^	383.4 ± 55.1^a^, ^b^	124.3 ± 13.9
STZ‐INS	126.6 ± 12.7	120.4 ± 8.6	90.3 ± 10.5	137.5 ± 6.7
ANOVA	*F* = 89.82, *p* < 0.0001	*F* = 113.7, *p* < 0.0001	*F* = 229.7, *p* < 0.0001	*F* = 2.14, *p* = 0.152

^a^ Significant difference between control and STZ‐D groups. ****p < 0.0001.

^b^ Significant difference between STZ‐D and STZ‐INS groups. ^####^ *p* < 0.0001.

Neonatal BGCs measured on the day of birth (P0) revealed significantly higher levels in pups born to diabetic mothers compared to the other groups (****p < 0.0001 vs *control*, ^*####*^ *p* < 0.0001 vs *STZ‐INS*), demonstrating the persistence of maternal hyperglycemia in offspring. However, by 2 weeks postpartum (P14), neonatal BGCs showed a substantial decrease (****p < 0.0001), and no significant differences were observed among the groups at this time point.

### 3.2 Newborn Body and Brain Weights

In this study, the whole pups' body and brain weights were measured 14 days after birth, which was depicted in Table 3. Our statistical analysis illustrated a considerable reduction in the weights of the body and brain of pups born to diabetic mothers in comparison with STZ‐INS and control groups (**p < 0.01, *p < 0.05, respectively). These findings highlight the adverse effects of maternal diabetes on the growth and development of offspring.

**TABLE 3:** This table represents newborn body and brain weights (g). Our data showed a significant decrease in the neonatal body and brain weights in STZ‐D when compared with other groups (*p* < 0.01, *p* < 0.05, respectively). Mean ± SD.

Groups	Control	STZ‐D	STZ‐INS	ANOVA
Body weight (g), *n* = 10	26.60 ± 0.75	23.07 ± 2.06 ^a,b^	26.03 ± 1.12	*F* = 11.61, *p* = 0.0007
Brain weight (g), *n* = 10	0.88 ± 0.05	0.81 ± 0.04 ^c,d^	0.87 ± 0.02	*F* = 5.65, *p* = 0.0148

*Note:* a, c: Significant difference between Control and STZ‐D groups. a: ****p < 0.0001, c: *p < 0.05. b, d: Significant difference between STZ‐D and STZ‐INS groups. b: ^####^ *p* < 0.0001, d: *p < 0.05.

### 3.3 Effects of Maternal Diabetes on the Number of Dark Neurons

**FIGURE 1:**
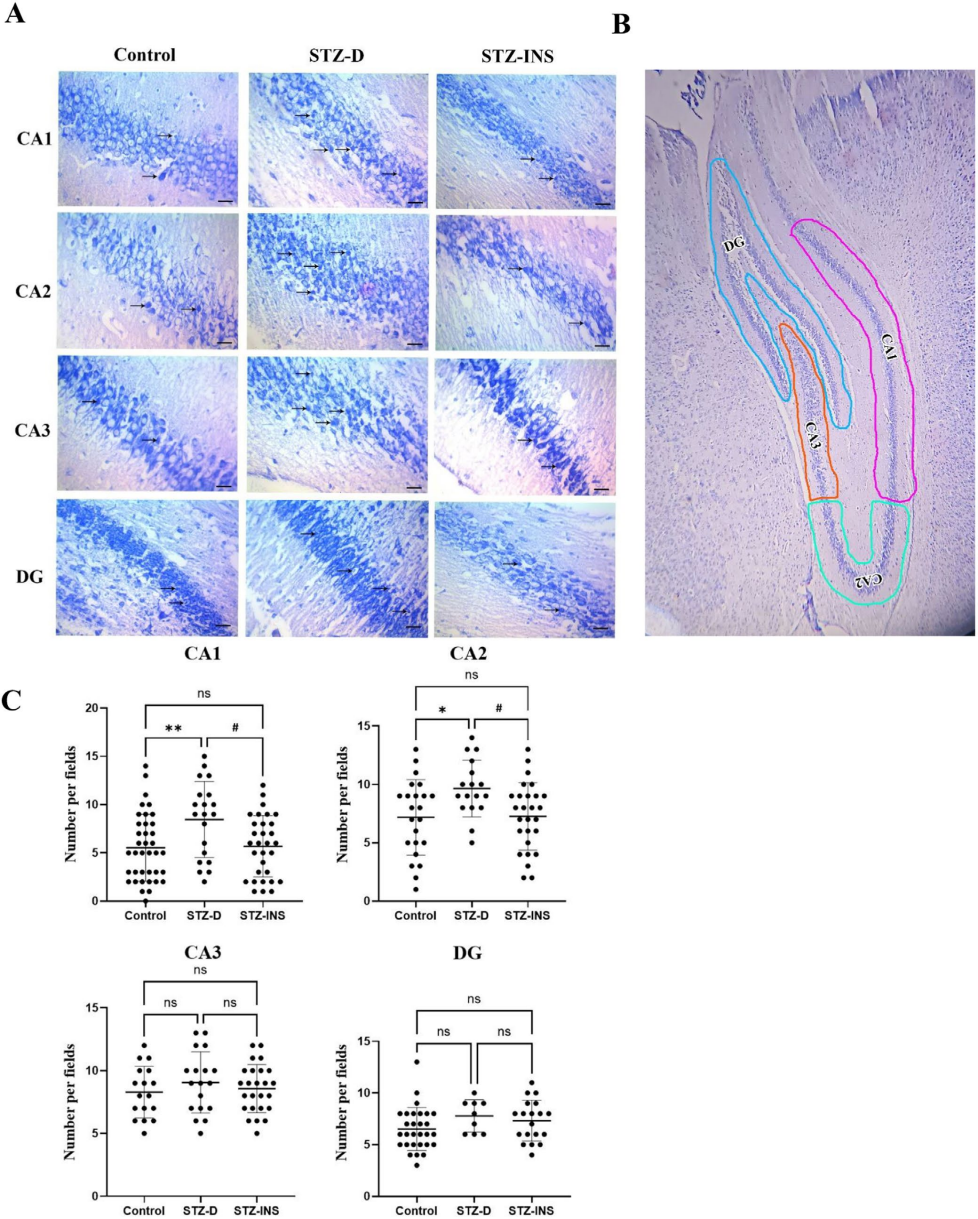
(A, B) Micrographs show toluidine blue staining of the neonatal hippocampus in all experimental groups at P14. Black arrows indicate dark neurons. (Scale bare: 25 μm). (C) Given graphs depict the effects of maternal diabetes on the number of dark neurons in different hippocampal subfields in all three groups. According to our findings, the number of dark neurons in the CA1 and CA2 hippocampal subfields was significantly higher in the STZ‐D group than in the STZ‐INS and control groups. Twenty fields were counted for each hippocampal subfield. (*, ^#^ *p* < 0.05, **p < 0.01). Mean ± SD.

To evaluate the neuronal damage, toluidine blue staining was carried out and the number of dark neurons was counted (Figure 1A,B). Researchers determined the various hippocampal subregions (CA1, CA2, CA3, DG) by using the rat brain atlas by Paxinos and Watson [26]. Histological sections were examined under a light microscope (5 rats/group, 4 fields/animal, total 20 fields), revealing a significant increase in the number of dark neurons in the CA1 and CA2 hippocampal subfields of offspring born to diabetic mothers compared to the other two experimental groups (*, ^#^ *p* < 0.05, **p < 0.01). In contrast, according to our results, there were no appreciable variations in the number of dark neurons among the three experimental groups in the CA3 and DG subfields (Figure 1C).

### 3.4 Effects of Maternal Diabetes on Hippocampal Volume

**FIGURE 2:**
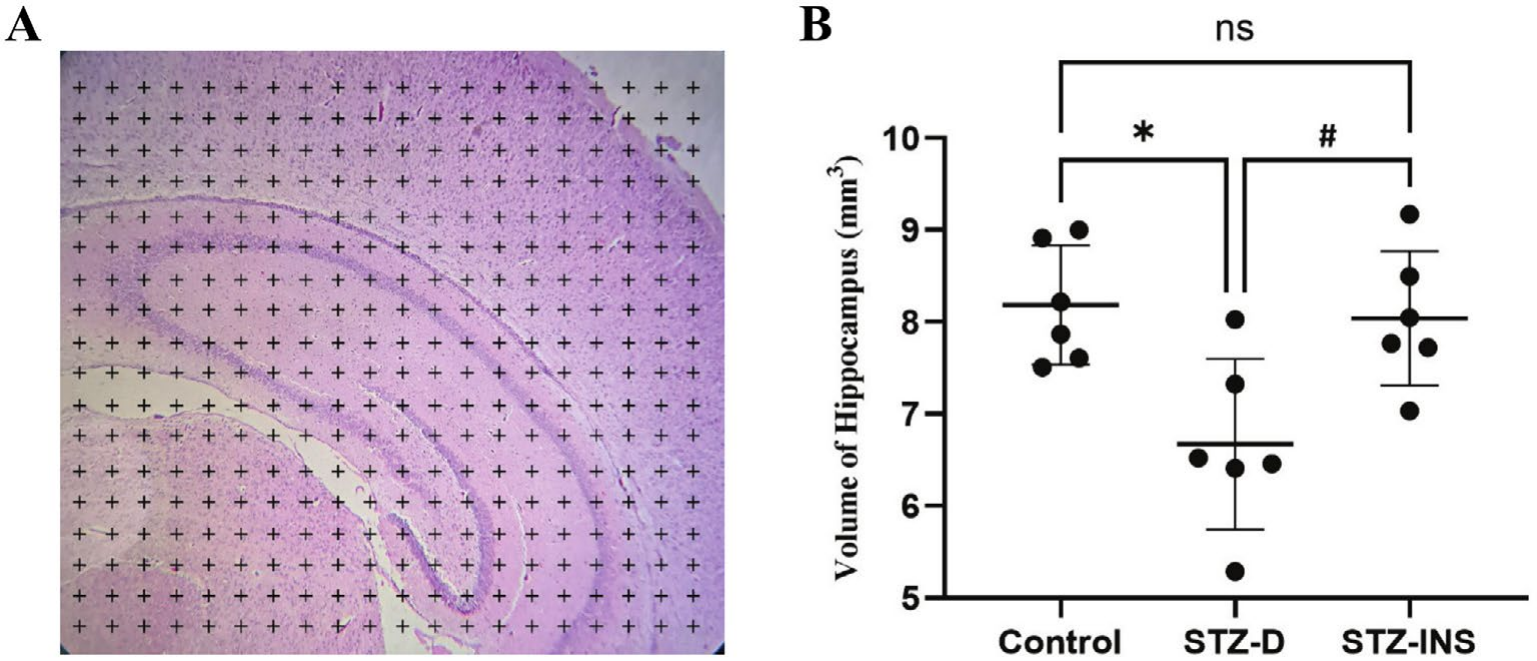
(A) Overlaying a grid of dots on the histological picture of the hippocampus to estimate newborns' hippocampal volume. (B) Given graph illustrates the effects of maternal diabetes on the volume of the hippocampus in all three experimental groups. Statistical analysis shows a significant decrease in the hippocampal volume of pups born to diabetic mothers when compared to other groups (*, ^#^ *p* < 0.05). Mean ± SD. (6 rats/groups, 10 sections/animal).

The stereological estimation of hippocampal volume was performed using H&E‐stained sections (Figure 2A). Results showed a significant reduction in hippocampal volume in neonatal rats of the STZ‐D group compared to the STZ‐INS and control groups (*, ^#^ *p* < 0.05, Figure 2B). This finding suggests that maternal diabetes leads to structural impairments in the hippocampus.

### 3.5 Effects of Maternal Diabetes on Spatial Distribution of Neurons in the Neonatal Hippocampus

**FIGURE 3:**
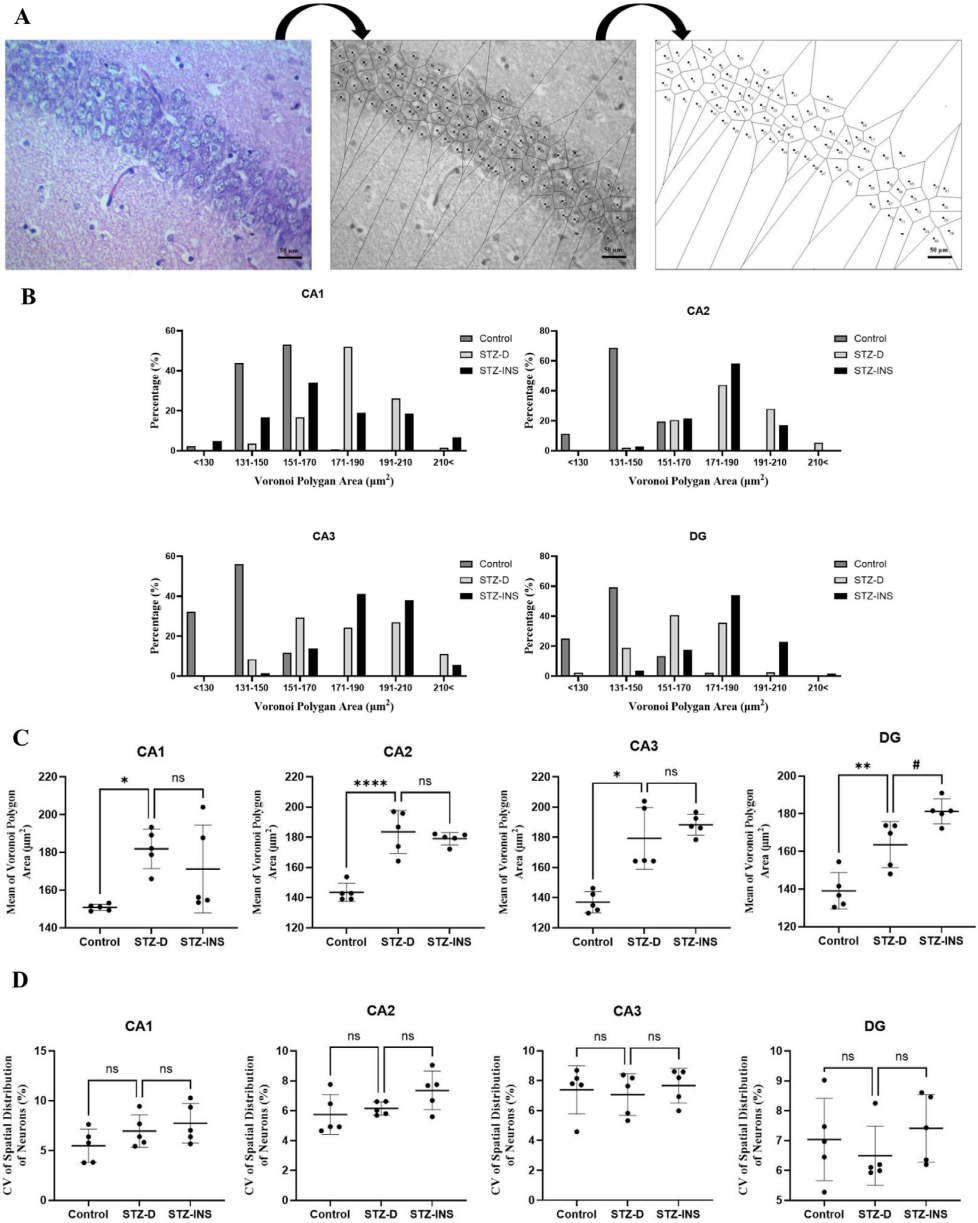
(A) Representative photograph, polygon area, and schematic of Voronoi tessellation of the neuron in the CA1 hippocampal subfield in the control group. Following scale calibration, neuronal nuclei were identified and overlaid with polygons using ImageJ software, enabling the visualization and analysis of neuronal spatial distribution. (B) The distribution of the Voronoi polygon areas (μm^2^). (C) The mean of polygon areas (μm^2^). The coefficient of variation (CV) of the spatial distribution of neurons in different groups: All experimental groups had a regular distribution (CV less than %33). (*, ^#^ *p* < 0.05, **p < 0.01, ****p < 0.0001). (*n* = 5 rats/group).

Figure 3A shows a photograph and schematic of the Voronoi polygons area for neonatal hippocampus sections. According to polygon area distribution, the data show about 97% of the polygon's areas of the neurons in the CA1 in the control group were located in the range of 131–170 μm^2^ and this was 20% and 51% in the STZ‐D and STZ‐INS, respectively. Moreover, in the diabetic animals, about 78% of the polygon areas were placed in the range of 171–210 μm^2^, while about 37.5% and 0.7% of polygon areas were located in this range in the STZ‐INS and control groups, respectively. It shows that this distribution in diabetic animals was shifted to the right (Figure 3B).

In the CA2 subfield, about 88% of the polygon's areas of the neurons were situated in the range of 131–170 μm^2^ in the control animals, while in the STZ‐D and STZ‐INS groups, about 22.5% and 24% of the polygon's areas were located in this range. Furthermore, about 72% of the polygon's areas were placed in the range of 171–210 μm^2^ in the diabetic group, while in the STZ‐INS and control groups, about 75% and 1% were observed in this range. In fact, the distribution of the polygon's areas was shifted to the right in the diabetic animals (Figure 3B).

Regarding the CA3 subregion, our data revealed that about 88% of the polygon's areas of the neurons were located in the range of > 130 to 150 μm^2^ in the hippocampus of the control group, and this was 8.5% and 1.5% of the polygon's areas in the STZ‐D and STZ‐INS groups, respectively. In addition, about 53.5% of the polygon's areas were placed in the range of 151–190 μm^2^ in the neonate born to diabetic mothers, while it was about 54.8% and 11.7% of the polygon's areas that were placed in this range in the STZ‐INS and control groups, respectively. It represents that in the diabetic animals the distribution of the polygon's areas was shifted to the right (Figure 3B).

Based on our data, in the DG, about 85% of the polygon's areas of the neurons were placed in the range of > 130 to 150 μm^2^ in the control group, whereas it was 21.2% and 3.7% of the polygon's areas in the STZ‐D and STZ‐INS groups, respectively. Moreover, about 76.5% of the polygon's areas were placed in the range of 151–190 μm^2^ in the neonate born to diabetic mothers, while about 71.5% and 15.7% of the polygon's areas was placed in this range in the STZ‐INS and control groups, respectively. It demonstrates that in the diabetic animals, the distribution of the polygon's areas was shifted to the right (Figure 3B).

According to our data, the area that a neural cell occupies or the mean polygon area is significantly higher in the hippocampus of newborns born to diabetic mothers in comparison to control pups in all hippocampal subregions (CA1, CA2, CA3, DG) (*p < 0.05, ****p < 0.0001, *p < 0.05, **p < 0.01, respectively) (Figure 3C).

Furthermore, the mean coefficient of variance (CV) for the polygon area is presented in Figure 3D. In all experimental groups, the CV values across the hippocampal subregions remained below 33%. These findings indicate that while maternal diabetes led to an increase in CV, the overall neuronal distribution within all hippocampal subfields showed a regular pattern.

### 3.6 Effects of Maternal Diabetes on Expression of Autophagy‐Related Genes in the Newborns' Hippocampus

**FIGURE 4:**
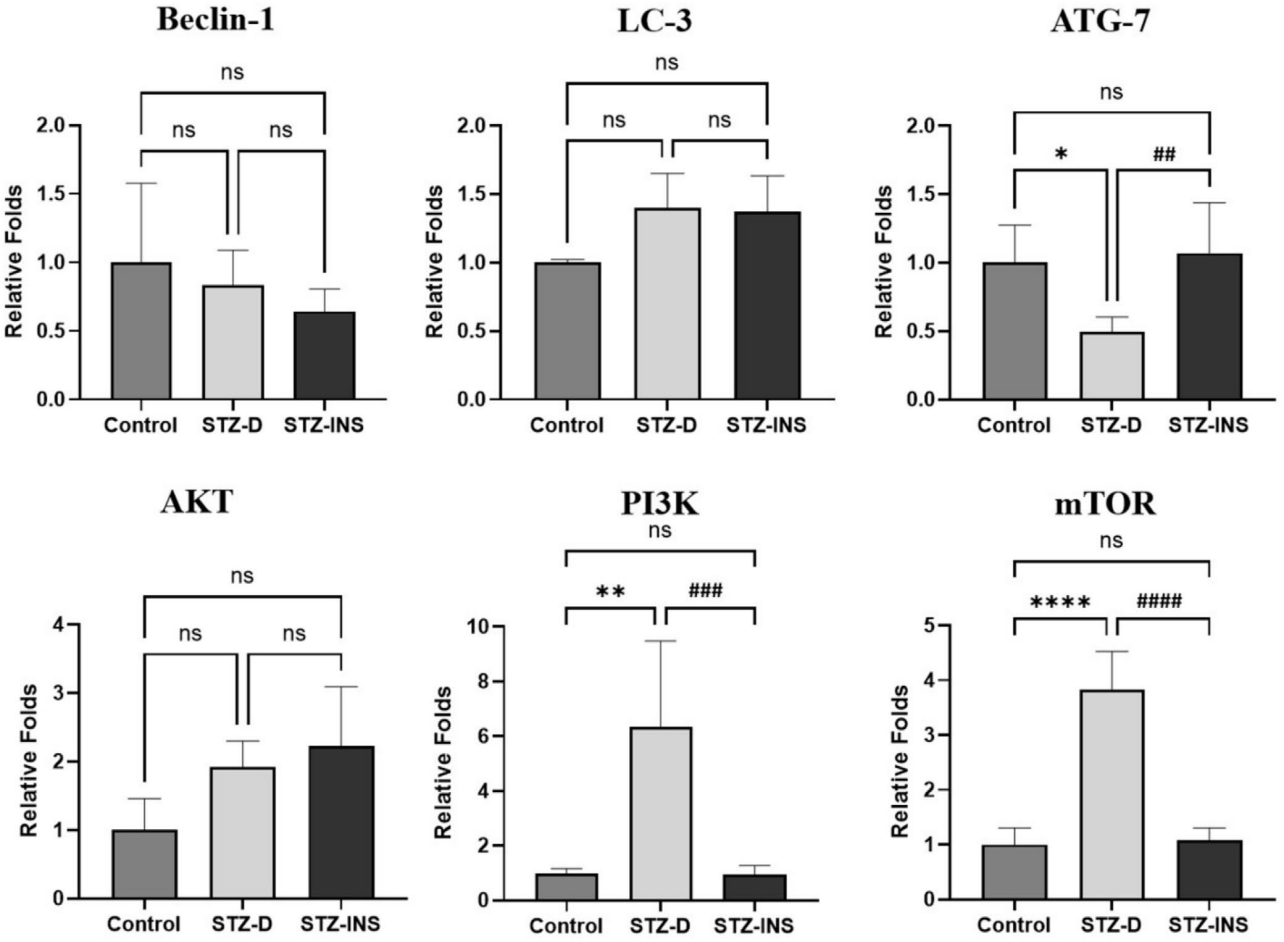
Given graphs illustrate the effects of maternal diabetes on the mRNA expression (Mean ± SD) of Beclin‐1, LC‐3, and ATG‐7, as well as AKT/PI3K/mTOR, in the hippocampus of rat newborns in all three experimental groups. Our results revealed a marked downregulation in the ATG‐7 and significant upregulation in the PI3K and mTOR gene expression in the hippocampus of pups born to diabetic mothers when compared to other groups (*p < 0.05; **p < 0.01; ****p < 0.0001; ^##^ *p* < 0.01; ^###^ *p* < 0.001; ^####^ *p* < 0.0001). (*n* = 6).

Expression levels of autophagy‐related genes (Beclin‐1, LC3, ATG‐7) and components of the AKT/PI3K/mTOR signaling pathway were assessed using RT‐PCR in the hippocampus of offspring at P14. The analysis demonstrated a significant decrease in ATG‐7 expression in the STZ‐D group compared to the other groups (*p < 0.05 vs *control*, ^*##*^ *p* < 0.01 vs *STZ‐INS*), while Beclin‐1 and LC3 expression levels remained unchanged (Figure 4). In addition, as shown in Figure 4, mRNA expression of PI3K and mTOR was markedly upregulated in the hippocampus of pups born to diabetic mothers compared to the control and STZ‐INS groups 2 weeks after birth ([**p < 0.01 vs. *control*, ^*###*^ *p* < 0.001 vs. *STZ‐INS*], [****p < 0.0001 vs. *control*, ^*####*^ *p* < 0.0001 vs. *STZ‐INS*], respectively). Nevertheless, there was no statistically significant difference in the level of AKT gene expression between the STZ‐D group and the STZ‐INS and control groups.

## 4 Discussion

The present study highlights the adverse effects of maternal hyperglycemia during the pregnancy period and the role of treatment by insulin on neonatal hippocampal development, focusing on autophagy regulation and hippocampal morphology. Since it has been demonstrated that the first two postnatal weeks in rodents are extremely crucial for central nervous system development, and because they roughly correlate to the mid‐second to mid‐third trimester of human gestation, moreover, by this stage, neonatal blood glucose levels stabilize following early postnatal fluctuations, allowing us to assess the long‐term impact of maternal diabetes in a relatively steady metabolic environment. Hence, we concentrated on these weeks, and all histological and molecular analyses were performed at postnatal day 14 (P14) [11].

The findings align with previous research that associates gestational diabetes mellitus (GDM) with structural and functional deficits in the fetal central nervous system (CNS), particularly in the hippocampus, a critical region for learning and memory. Our results revealed that gestational diabetes could cause a significant reduction in hippocampal volume and also a marked increase in the number of damaged neurons (dark neurons) in the offspring of diabetic mothers. In addition, this glucose imbalance during pregnancy could influence the autophagy process by affecting the expression of related genes.

It is well‐documented that fetuses in diabetic mothers grow in completely different situations than in healthy mothers, which significantly affects fetal development, particularly the central nervous system, through mechanisms like maternal hyperglycemia and oxidative stress [6, 8, 9, 11]. These disturbances cause hypoxia, oxidative stress, and neuronal apoptosis, leading to growth restriction or low birth weight due to maternal vascular complications [5]. Aligned with these findings, our results showed a significant reduction in the neonatal body and brain weight in the STZ‐D group compared to other groups (Table 3).

The adverse impacts of diabetes during pregnancy on the developing fetal central nervous system are well established; however, the precise mechanisms are still to be elucidated [11, 12]. However, prior investigation revealed that maternal diabetes during pregnancy may cause fetal hyperglycemia, which in turn stimulates the proliferation of pancreatic β‐cells, resulting in fetal hyperinsulinemia. Additionally, it is well recognized that this hyperinsulinemia occurs in the final trimester and puts newborns at risk for early onset hypoglycemia after birth, which raises the possibility of CNS development impairment [9, 27, 28]. Animal studies clearly show that diabetes during pregnancy can cause structural and functional changes in the hippocampus and cerebellum of pups. These studies show that pups born to diabetic mothers have significantly lower hippocampal and cerebellar volume compared to healthy mothers [29, 30]. Additionally, stereological investigations revealed a notable reduction in the number of neural cells in the hippocampus of these animals. Our stereological evaluation demonstrated that the hippocampal volume of pups in the STZ‐D group is substantially lower than in other experimental groups, which is consistent with previous investigations. In previous studies, Sadeghi et al. [31] indicated that increases in neuronal apoptosis and declines in neurogenesis in the hippocampus owing to maternal diabetes can be considered a reason for the decrease in the volume of the hippocampus of newborns born to diabetic mothers.

It is well‐documented that some processes, including apoptosis, oxidative stress, autophagy, and mitochondrial dysfunction, play important roles in extending neural tissue damage in various neurodegenerative disorders [32, 33, 34, 35, 36]. In this regard, it is accepted that maternal diabetes during pregnancy can significantly increase apoptosis in the neonatal central nervous system, which leads to disruption of normal CNS development and reduced neuronal cell density. Previous studies indicate that diabetes during pregnancy may elevate pro‐apoptotic gene expression (Bax) while reducing anti‐apoptotic gene expression (Bcl‐2) in the hippocampus, potentially resulting in increased neural tissue damage and impairing neurodevelopmental processes [24, 37, 38].

Indeed, while insulin's glycemic regulatory function plays a significant role in mitigating the adverse effects of maternal diabetes, accumulating evidence supports the notion that insulin also exerts neuroprotective, anti‐apoptotic, and anti‐inflammatory effects in the central nervous system [39, 40]. Several studies have demonstrated that insulin signaling pathways, particularly the PI3K/AKT pathway, play critical roles in neuronal survival, synaptic plasticity, and neurogenesis. For example, the activation of the PI3K/AKT pathway by insulin has been shown to inhibit apoptosis through inhibition of pro‐apoptotic proteins (Bad, caspase‐9) and enhancement of anti‐apoptotic proteins (Bcl‐2) [41, 42]. This pathway also interacts with mTOR, which is implicated in both autophagy regulation and neuronal growth. In our study, the upregulation of PI3K and mTOR may reflect a compensatory response aimed at preserving neuronal viability in the context of insulin treatment. In addition, insulin exerts anti‐inflammatory effects in the central nervous system through multiple mechanisms [43]. It suppresses the NF‐κB signaling pathway, a key regulator of pro‐inflammatory cytokine expression, thereby reducing the production of mediators such as TNF‐α, IL‐1β, and IL‐6. Additionally, insulin attenuates the activation of microglia and astrocytes (central immune cells in the brain) effectively limiting neuroinflammatory responses [39, 44]. By enhancing neuronal insulin signaling, it also helps maintain the integrity of the blood–brain barrier and restricts leukocyte infiltration [43]. Collectively, these actions underscore insulin's potential as a neuroprotective and anti‐inflammatory agent in CNS‐related pathologies.

Our histological analysis revealed substantial neuronal damage in the CA1 and CA2 hippocampal subfields of offspring from diabetic mothers. These findings are consistent with previous studies suggesting that hyperglycemia‐induced oxidative stress and inflammation contribute to neuronal apoptosis. Furthermore, the preservation of hippocampal structure and reduced dark neurons in the insulin‐treated group suggest that effective glycemic control mitigates neuronal damage, emphasizing the critical role of maternal glucose management during pregnancy.

Nowadays, it is well documented that the spatial organization of neurons across different brain regions, along with variations in neuronal density and volume, plays a crucial role in developmental processes, connectivity patterns, and functional outcomes. These structural attributes influence neuronal positioning, contributing to brain formation and the establishment of neural circuits [25, 45]. Recent research has demonstrated that a variety of neurodevelopmental and neurodegenerative disorders may result in changes in brain cell density and spatial distribution [25, 46].

In addition to the increased number of dark neurons, we assessed neuronal spatial organization using Voronoi tessellation. The analysis showed a significant shift toward higher polygon areas in the diabetic group; however, the coefficient of variation (CV) across hippocampal subregions remained below 33%, indicating that the overall spatial distribution of neurons retained a regular pattern. This finding suggests that maternal diabetes did not disrupt the geometric arrangement of neurons, but may have influenced cell packing density or neuronal volume, leading to an increase in the area occupied by each neuron. Thus, the observed changes in polygon size likely reflect subtle morphometric alterations rather than a loss of spatial regularity. In this regard, a recent study found that cerebral ischemia may alter the normal spatial distribution of CA1 hippocampal pyramidal neurons, resulting in a more random pattern [47].

In this regard, a recent study found that cerebral ischemia may alter the normal spatial distribution of CA1 hippocampal pyramidal neurons, resulting in a more random pattern.

It has been shown that autophagy, along with other mechanisms like chaperone proteins, the ubiquitin‐proteasome system, and endoplasmic reticulum‐associated protein degradation, is essential for maintaining functional synapses and preventing neurodegeneration [48]. Autophagy, a cellular process that degrades and recycles damaged organelles and proteins, plays a crucial role in neuronal homeostasis, plasticity, and protein quality control, promoting cell survival and function, and regulating cell growth [17, 49]. It helps maintain a balance between cell proliferation and apoptosis during pregnancy and influences the maturation of neural stem cells into functional neurons [49]. Research suggests that disruptions in autophagic processes caused by maternal diabetes could lead to impaired CNS development and function [16, 48].

The results of recent investigations have revealed that the deletion of key autophagy‐related genes, such as ATG‐5 and ATG‐7, results in significant neuronal degeneration across various regions, including the cerebral cortex, cerebellum, hypothalamus, and retina. Neurons lacking these genes exhibit abnormal protein aggregation and progressive axonal dystrophy, leading to cell‐autonomous degeneration, particularly in Purkinje neurons of the cerebellum. These findings emphasize the essential function of autophagy in maintaining neuronal integrity and preventing the toxic accumulation of proteins [48, 50, 51, 52].

It is well documented that diabetes and hyperglycemic conditions can suppress autophagy through dysregulation of key signaling pathways, particularly the PI3K/AKT/mTOR and AMPK pathways. In high‐glucose environments, insulin or growth factors activate PI3K, leading to AKT phosphorylation and subsequent activation of mTOR (serves as a pivotal regulator of autophagy). Activated mTOR inhibits autophagy by phosphorylating and inactivating ULK1, a critical initiator of autophagosome formation. Concurrently, hyperglycemia‐associated metabolic excess reduces AMPK activation, which would otherwise promote autophagy by phosphorylating ULK1 at distinct activating residues. Therefore, in diabetic conditions, the dual effect of mTOR activation and AMPK inhibition synergistically suppresses autophagy initiation, impairing the clearance of damaged proteins and organelles and potentially contributing to neurodegenerative disease progression. This regulation highlights the critical role of mTOR in balancing cellular homeostasis [53, 54, 55].

At the molecular level, this study identified downregulation of ATG‐7 and upregulation of PI3K and mTOR gene expressions in the hippocampus of offspring from diabetic mothers. The alterations in these autophagy‐related genes suggest a disruption in the balance between cellular repair and degradation mechanisms. Activation of the PI3K/mTOR pathway likely exacerbates neuronal damage by inhibiting autophagy, a finding supported by existing literature on the neurotoxic effects of impaired autophagic flux. Interestingly, various studies reported that the autophagy process has a dual role in neurodegenerative disorders [56, 57, 58]. This highlights the complexity of autophagy regulation in response to metabolic stress and underscores the need for further exploration of its mechanistic pathways in the context of CNS development.

## 5 Conclusion

This study highlights the significant impact of maternal diabetes on neonatal hippocampal development, emphasizing its role in altering autophagy and causing structural damage to the central nervous system (CNS). The findings demonstrated that maternal hyperglycemia induces hippocampal volume reduction, increased neuronal damage, and dysregulated autophagy‐related gene expression, particularly through the activation of the PI3K/mTOR pathway. Importantly, insulin treatment during pregnancy mitigated these adverse effects, preserving hippocampal structure and reducing neuronal damage. These results underscore the critical importance of glycemic control during pregnancy in preventing neurodevelopmental impairments associated with gestational diabetes mellitus (GDM). Furthermore, the study provides new insights into the mechanistic pathways involved, such as autophagy dysregulation, which could serve as potential therapeutic targets. Future research should focus on exploring long‐term cognitive outcomes and identifying interventions to enhance CNS development in the offspring of diabetic mothers.

## 6 Limitations

While this study provides valuable insights into the early effects of maternal diabetes on hippocampal development and autophagy‐related gene expression, several limitations should be considered. First, all analyses were performed at postnatal day 14 (P14), which corresponds to the third trimester of human gestation in rodent models. Although this time point is well‐suited for studying early neurodevelopmental changes, the absence of long‐term follow‐up prevents us from determining whether the observed alterations persist or result in functional deficits during later stages of brain maturation. Second, the study did not include behavioral assessments, such as learning or memory tests, which would help to establish the functional significance of the structural and molecular changes identified in the hippocampus. Third, gene expression data were normalized using a single housekeeping gene (β‐actin). While this gene is widely used, relying on a single reference gene may not adequately account for variability under experimental conditions such as maternal diabetes. Incorporating multiple validated housekeeping genes in future work would strengthen the reliability of gene expression results. To address these limitations, future studies should include extended developmental time points and behavioral evaluations to better assess the long‐term impact of maternal diabetes on central nervous system function.

## Author Contributions

Conceptualization: Saeed Vafaei‐Nezhad. Data Curation: Saeed Vafaei‐Nezhad, Saleheh Mansouri Boutegaz. Formal Analysis: Mohammad Reza Namavar, Mehri Shadi. Investigation: Saeed Vafaei‐Nezhad, Mehri Shadi, Saleheh Mansouri Boutegaz. Methodology: Saeed Vafaei‐Nezhad, Mehri Shadi, Mohammad Reza Namavar, Hamid Kabiri‐rad. Project Administration: Saeed Vafaei‐Nezhad. Supervision: Saeed Vafaei‐Nezhad, Mehri Shadi. Validation: Saeed Vafaei‐Nezhad. Writing – Original Draft: Saeed Vafaei‐Nezhad, Mehri Shadi, Mohammad Reza Namavar, Saleheh Mansouri Boutegaz. Writing – Review and Editing: Saeed Vafaei‐Nezhad, Mehri Shadi.

## Conflicts of Interest

The authors declare no conflicts of interest.

## Supporting information


Appendix S1.

## Data Availability

Research data are not shared.
