# Supplementary material for: Neurodevelopmental Consequences of Maternal Diabetes: Autophagy and Spatial Arrangement of Hippocampal Neurons
Source: CNS Neurosci Ther. 2025 Jul 14;31(7):e70518. doi: 10.1111/cns.70518 (PMC12260216; doi:10.1111/cns.70518)
Supplement: Supplementary file 1 — Appendix S1. [file CNS-31-e70518-s001.pptx]

## Slide 1
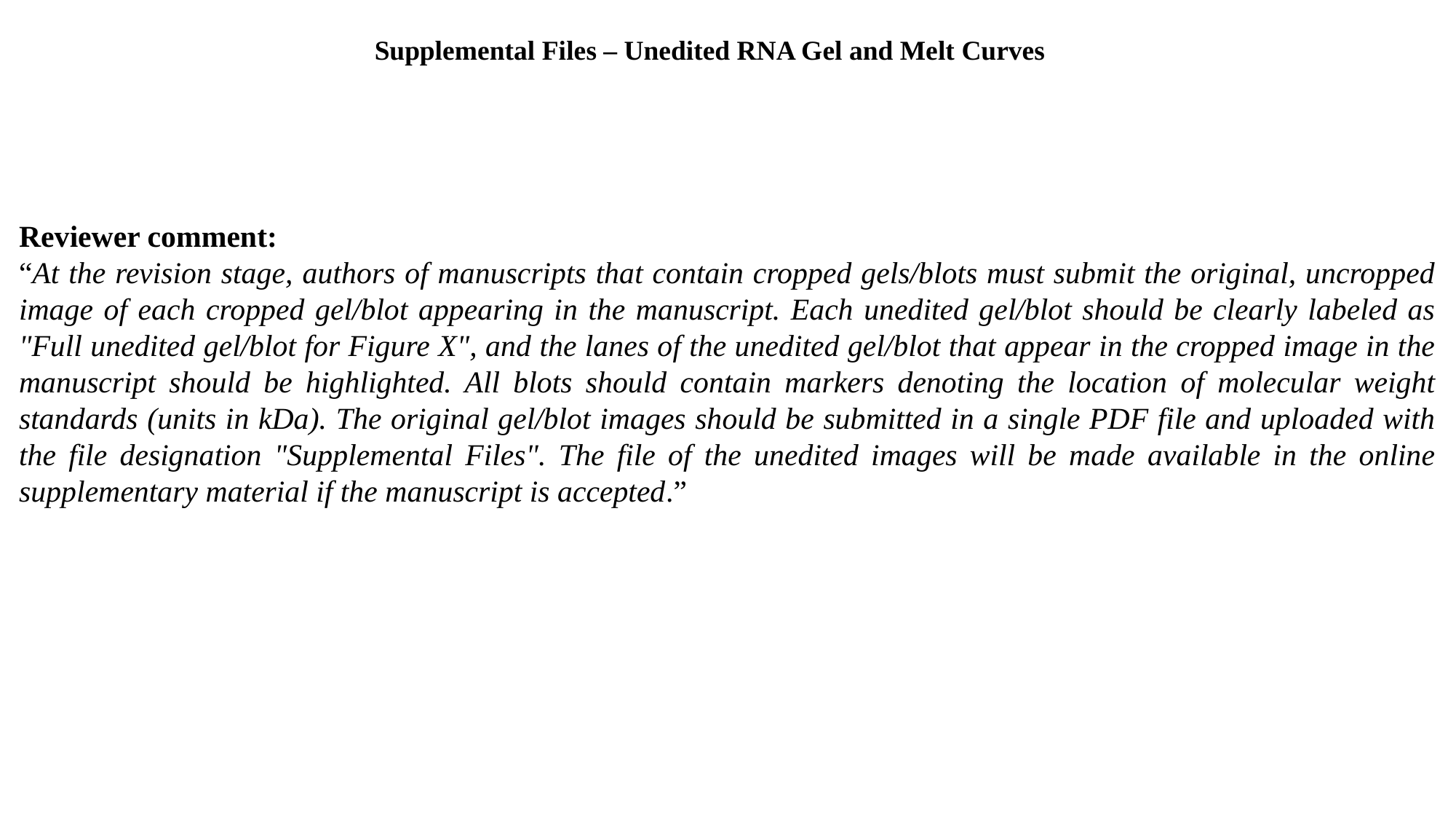

Supplemental Files – Unedited RNA Gel and Melt Curves
Reviewer comment:
“At the revision stage, authors of manuscripts that contain cropped gels/blots must submit the original, uncropped image of each cropped gel/blot appearing in the manuscript. Each unedited gel/blot should be clearly labeled as "Full unedited gel/blot for Figure X", and the lanes of the unedited gel/blot that appear in the cropped image in the manuscript should be highlighted. All blots should contain markers denoting the location of molecular weight standards (units in kDa). The original gel/blot images should be submitted in a single PDF file and uploaded with the file designation "Supplemental Files". The file of the unedited images will be made available in the online supplementary material if the manuscript is accepted.”

## Slide 2
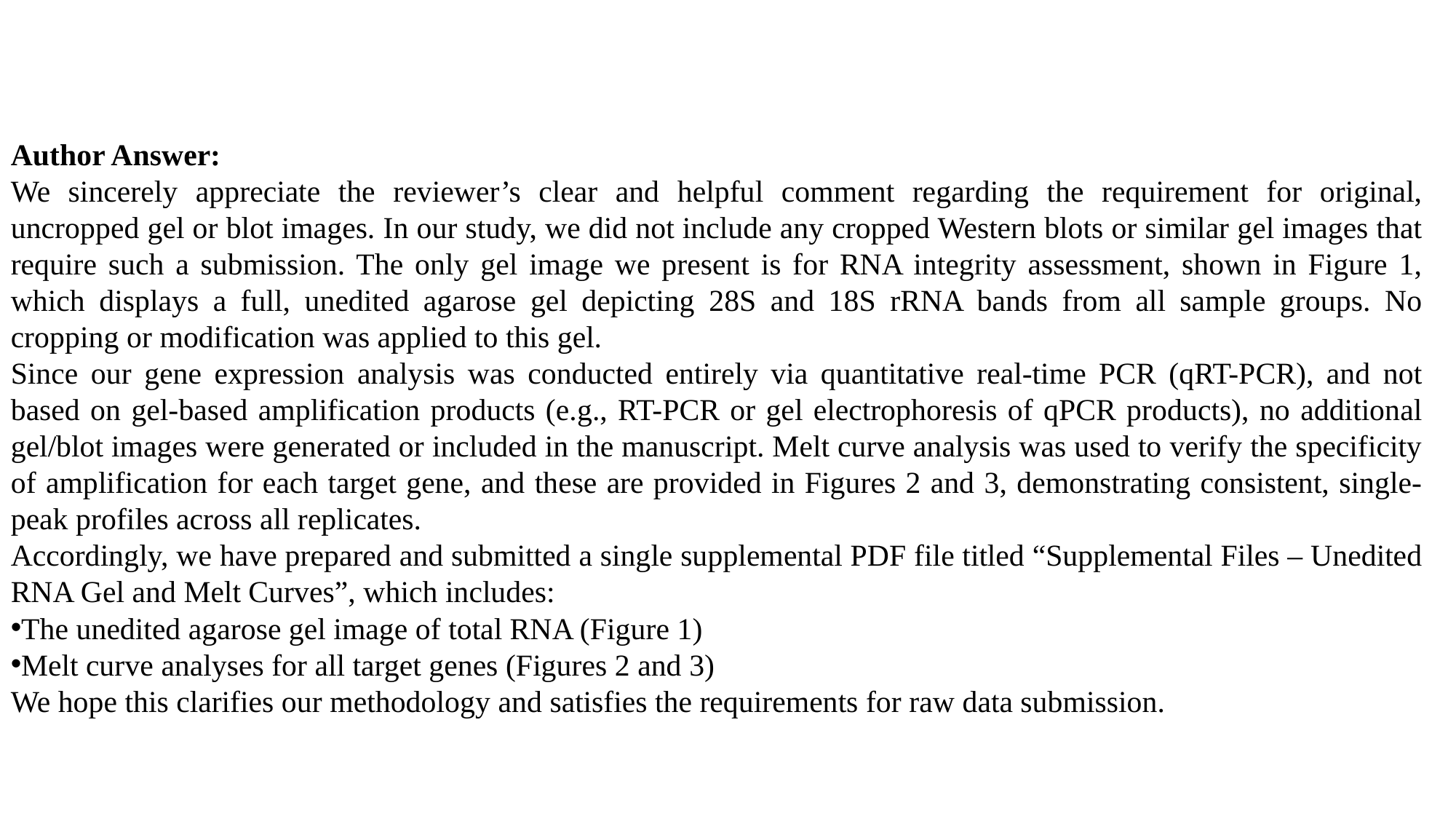

Author Answer:
We sincerely appreciate the reviewer’s clear and helpful comment regarding the requirement for original, uncropped gel or blot images. In our study, we did not include any cropped Western blots or similar gel images that require such a submission. The only gel image we present is for RNA integrity assessment, shown in Figure 1, which displays a full, unedited agarose gel depicting 28S and 18S rRNA bands from all sample groups. No cropping or modification was applied to this gel.
Since our gene expression analysis was conducted entirely via quantitative real-time PCR (qRT-PCR), and not based on gel-based amplification products (e.g., RT-PCR or gel electrophoresis of qPCR products), no additional gel/blot images were generated or included in the manuscript. Melt curve analysis was used to verify the specificity of amplification for each target gene, and these are provided in Figures 2 and 3, demonstrating consistent, single-peak profiles across all replicates.
Accordingly, we have prepared and submitted a single supplemental PDF file titled “Supplemental Files – Unedited RNA Gel and Melt Curves”, which includes:
The unedited agarose gel image of total RNA (Figure 1)
Melt curve analyses for all target genes (Figures 2 and 3)
We hope this clarifies our methodology and satisfies the requirements for raw data submission.

## Slide 3
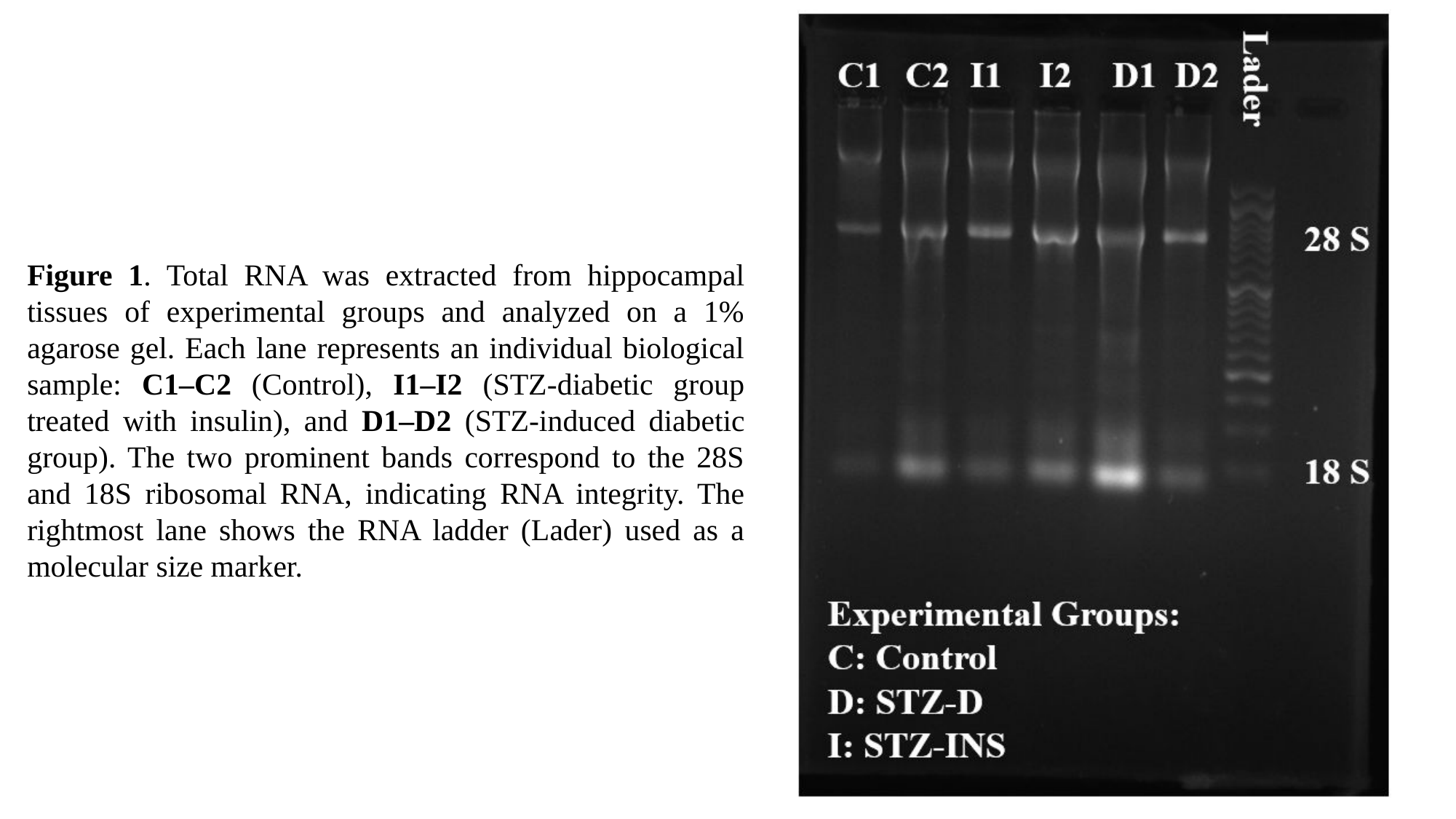

Figure 1. Total RNA was extracted from hippocampal tissues of experimental groups and analyzed on a 1% agarose gel. Each lane represents an individual biological sample: C1–C2 (Control), I1–I2 (STZ-diabetic group treated with insulin), and D1–D2 (STZ-induced diabetic group). The two prominent bands correspond to the 28S and 18S ribosomal RNA, indicating RNA integrity. The rightmost lane shows the RNA ladder (Lader) used as a molecular size marker.

## Slide 4
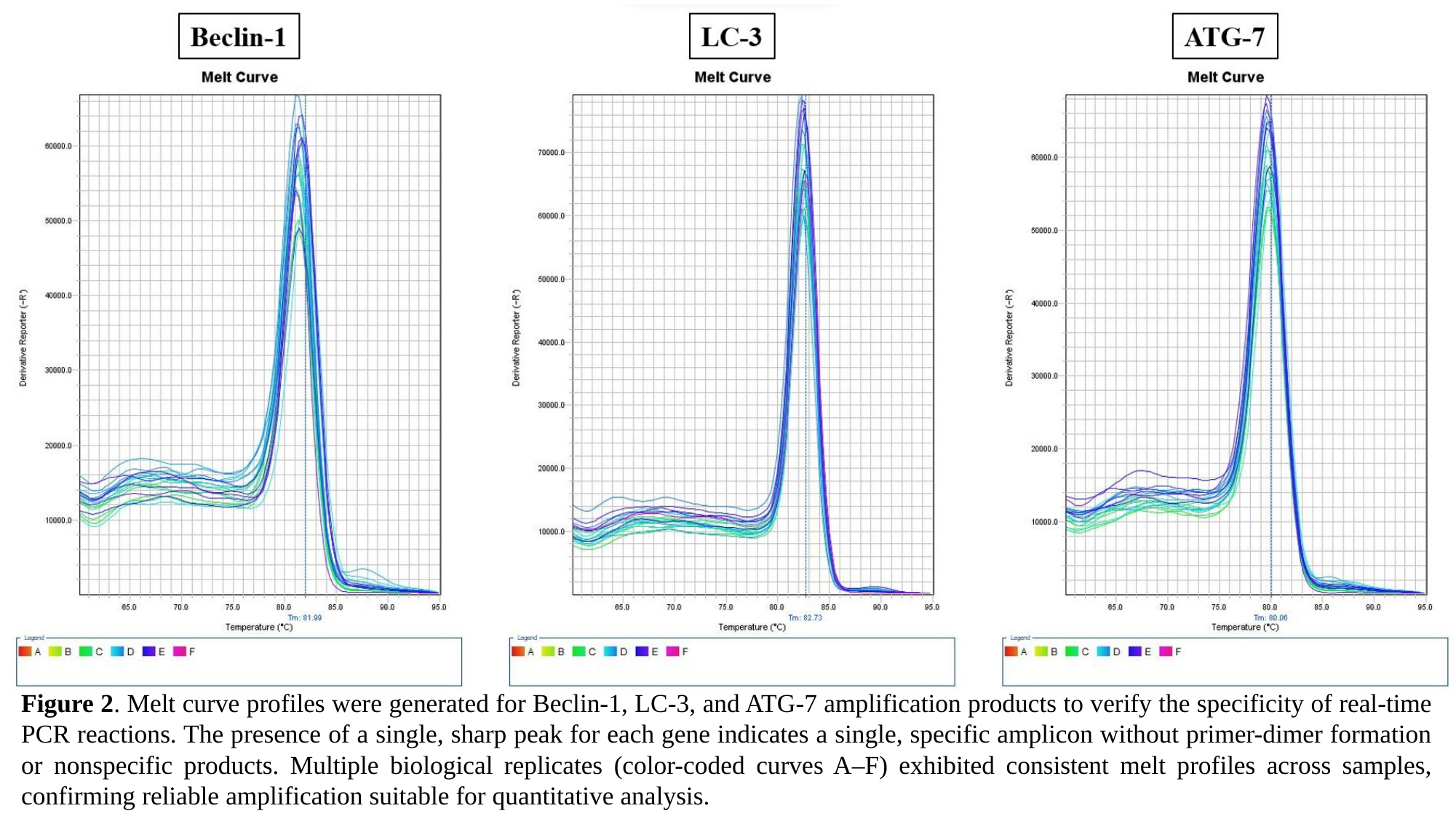

Figure 2. Melt curve profiles were generated for Beclin-1, LC-3, and ATG-7 amplification products to verify the specificity of real-time PCR reactions. The presence of a single, sharp peak for each gene indicates a single, specific amplicon without primer-dimer formation or nonspecific products. Multiple biological replicates (color-coded curves A–F) exhibited consistent melt profiles across samples, confirming reliable amplification suitable for quantitative analysis.

## Slide 5
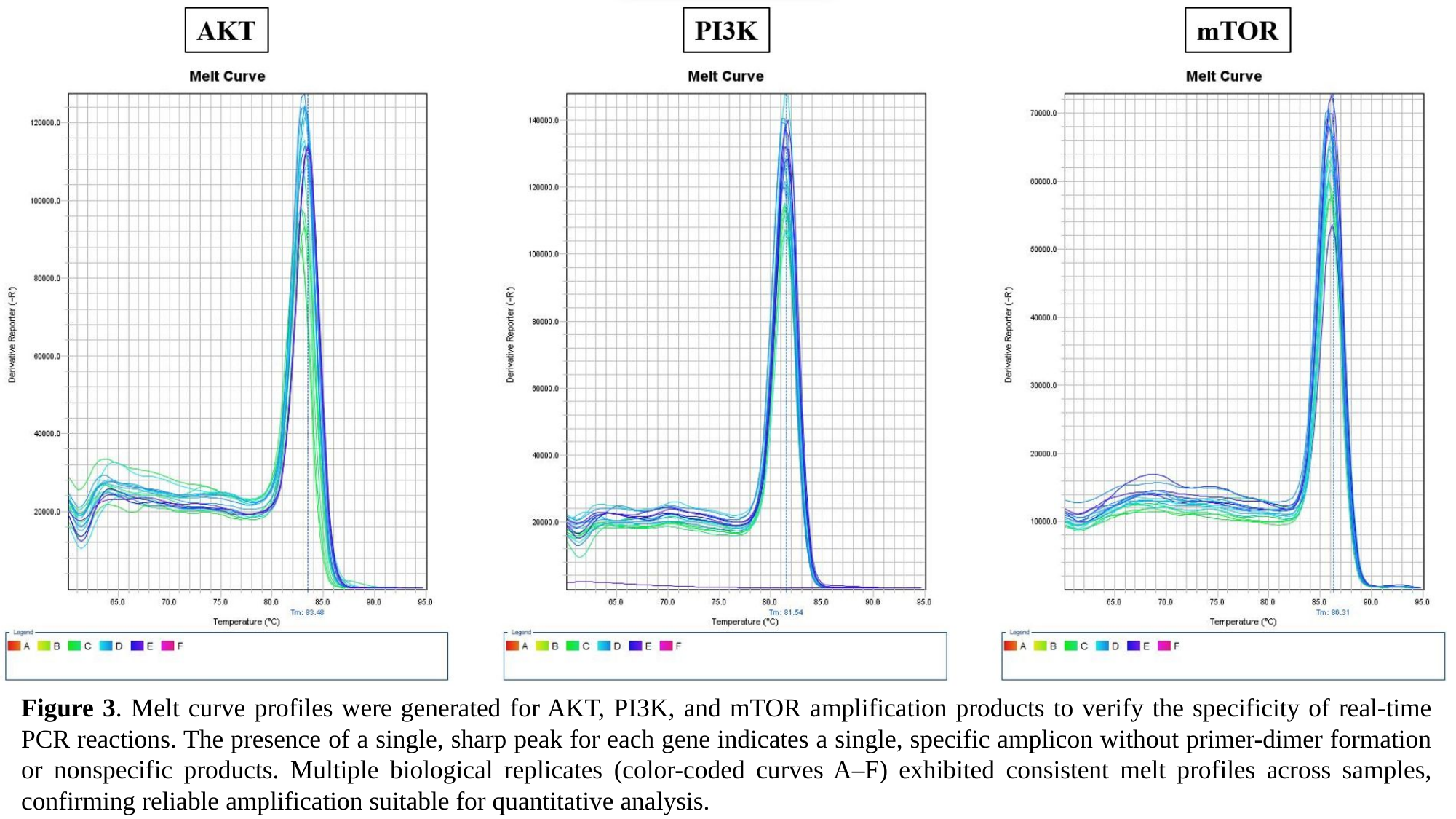

Figure 3. Melt curve profiles were generated for AKT, PI3K, and mTOR amplification products to verify the specificity of real-time PCR reactions. The presence of a single, sharp peak for each gene indicates a single, specific amplicon without primer-dimer formation or nonspecific products. Multiple biological replicates (color-coded curves A–F) exhibited consistent melt profiles across samples, confirming reliable amplification suitable for quantitative analysis.
